# Longitudinal dynamics and treatment-associated determinants of interferon-gamma responses in tuberculosis: a 10-year retrospective QuantiFERON-TB Gold study

**DOI:** 10.3389/fcimb.2026.1823104

**Published:** 2026-08-05

**Authors:** Chi Yang, Xiang Shi, Shaojun Zhang, Haili Hu, Yidian Liu, Xiaohui Hao, Wei Sha

**Affiliations:** Shanghai Clinical Research Center for Tuberculosis, Shanghai Pulmonary Hospital, Tongji University School of Medicine, Shanghai, China

**Keywords:** biomarker variability, diabetes mellitus, longitudinal dynamics, QuantiFERON-TB gold, tuberculosis treatment monitoring

## Abstract

Monitoring treatment efficacy and detecting recurrence are critical in tuberculosis (TB) management. However, longitudinal patterns of interferon-gamma release assay (IGRA) results and the quantitative influence of factors such as treatment remain poorly characterized, leaving its long-term performance unclear. This study aimed to investigate longitudinal changes in IGRA and explore their potential utility for monitoring immune dynamics and treatment response. We retrospectively examined 491 patients with two QuantiFERON-TB Gold tests (≥1-month interval) between 2013 and 2024. Analyses included moving average plots, linear regression, and stratification by time interval, treatment status, and treatment response. Multivariable regression and receiver operating characteristic analyses were performed to identify significant predictors. Longitudinal analysis revealed a significant but weak negative correlation between interferon-gamma (IFN-γ) levels and time, with an estimated decline of −0.33 IU/year (r = −0.17, P = 0.0001), albeit with poor model fit (R^2^ = 0.03, relative root mean square error = 16.33%). Moving average trajectories showed significant differences in changes in IFN-γ levels between treated and untreated subgroups (mean: −0.89 IU/mL, P < 0.001) and between therapy-converted and refractory subgroups (mean: 0.82 IU/mL, P < 0.0001). However, receiver operating characteristic analyses indicated poor discriminative power for these clinical statuses (area under the curve ≤ 0.582). Multivariable regression analysis confirmed diabetes mellitus as an independent factor associated with IFN-γ decreases (coefficient: −1.082, P = 0.040), alongside anti-TB treatment and the testing interval. The waning of IGRA reactivity was evident during TB and post-TB phases. However, despite significant associations with treatment status and treatment response, substantial variability limits its clinical utility. Therefore, QFT trends require cautious interpretation, driving the need for a more reliable assay or superior biomarkers.

## 1 Introduction

Tuberculosis (TB) remains a major global health concern, with over 10 million new cases and 1.25 million deaths reported in 2023 (World Health Organization, 2024a, 2024). The World Health Organization has set a target to eradicate TB by 2035, aiming for a 90% reduction in new cases and a 95% reduction in TB-related deaths relative to 2015 levels (World Health Organization, 2023). To achieve this goal, the World Health Organization-recommended integrated approach for TB elimination emphasizes the critical importance of early diagnosis during the TB stage, combined with the administration of preventive therapy for *Mycobacterium tuberculosis* (Mtb) infection—highlighting the urgent need for novel diagnostic tools to address the limitations of current methods across the disease spectrum (World Health Organization, 2024b; Li et al., 2025).

TB is increasingly recognized as a continuous spectrum of disease, extending beyond latent infection and active disease to include a distinct post-TB phase (Drain et al., 2018). However, despite successful treatment of TB, this subsequent phase is characterized by a significant and lasting health burden, as evidenced by post-TB sequelae accounting for nearly half of the annual disability burden associated with TB (58 million disability-adjusted life-years) (Menzies et al., 2021) and a standardized mortality ratio in TB survivors that is approximately three times higher than that in the general population (Romanowski et al., 2019). In direct response to these substantial clinical consequences, the expert consensus from the 2nd International Post-Tuberculosis Symposium: Life After TB underscored the critical importance of focused research in this phase, culminating in the proposal of a standardized case definition for a new clinical entity: post-TB lung disease. This entity refers to lung disease occurring after treated pulmonary or pleural TB and is defined by impairment in at least two of three domains—lung function, symptoms, or imaging—attributable to prior TB (Bisson et al., 2025). During the TB disease stage, monitoring treatment efficacy to determine the optimal treatment duration and accurately detecting TB recurrence during follow-up are equally vital for improving patient outcomes and ending the global TB epidemic, warranting comparable attention and the development of corresponding biomonitoring markers.

The interferon-gamma release assay (IGRA) is an immunodiagnostic test that quantifies T-cell-derived interferon-gamma (IFN-γ) production in response to Mtb-specific antigen stimulation *in vitro*. This measurement reflects the host’s cell-mediated immune response to Mtb (Lalvani, 2007; Pai et al., 2008; Diel et al., 2010; Mazurek et al., 2010; Pagaduan and Altawallbeh, 2023). In clinical practice, detection of IFN-γ released by sensitized peripheral blood lymphocytes serves as the basis for identifying TB, including Mtb infection (Andersen et al., 2000; Lalvani, 2007; Diel et al., 2010; Mazurek et al., 2010; Pelzer et al., 2025). The assay’s underlying principle is supported by human and animal studies demonstrating a correlation between the magnitude of antigen-specific T-cell responses and the extent of bacterial antigen exposure (Andersen et al., 2000). Due to its superior specificity and sensitivity compared to the tuberculin skin test, the IGRA has become a key laboratory method for diagnosing Mtb infection (Diel et al., 2010).

The IGRA is primarily employed for the diagnosis of Mtb infection and TB. Clinical evidence, however, suggests a correlation between bacterial load and the magnitude of the Mtb antigen-specific IFN-γ response (Ewer et al., 2006). This association implies that the intensity of the IFN-γ response may exhibit longitudinal dynamics during disease progression or following therapeutic intervention, supporting the utility of the IGRA in monitoring treatment efficacy and detecting disease recurrence.

Expanding the application of the IGRA for these purposes is constrained by two principal limitations. First, IGRA conversion and reversion patterns over time remain poorly defined (Horsburgh, 2023), and the clinical implications of these fluctuations are not fully elucidated (Pai and O’Brien, 2007). It has been generally assumed that positive tuberculin skin test (TST) or IGRA results typically persist for life, although it has been estimated that the reversion rate for TST positivity is approximately 10% per year (Daniels et al., 1948). This notion profoundly influences TB clinical management and epidemiological modeling, which often relies on the assumption of stable positivity rates. Consequently, IGRA reversion (from positive to negative) is a rare occurrence, with its significance frequently minimized or overlooked. Common approaches in the literature include emphasizing the stability of positive results in most individuals, selectively citing studies from high-prevalence settings to underscore its rarity, or dismissing early reversion as a “transient conversion” of limited clinical importance (Havlir et al., 1991; Menzies, 1999; Seo et al., 2014; Behr et al., 2019; Meier and Enders, 2021). Such interpretations often attribute reversion to biological variation or assay variability (Zhang et al., 2019; Mpande et al., 2021; Pérez-Recio et al., 2023). Nonetheless, accumulating evidence increasingly challenges this perspective.

As highlighted in the systematic review by Dale et al. (2024), the immune response to Mtb can wane over time. Validating and integrating this phenomenon into the current understanding of TB is crucial, as it necessitates a critical re-evaluation of clinical interpretation, epidemiological models, and public health strategies. The second principal limitation pertains to factors associated with longitudinal variability in IGRA results, particularly the influence of anti-TB treatment and its response, which have not been thoroughly characterized. Although the identification of biomarkers for monitoring TB treatment response represents a crucial goal for global disease control (Goletti et al., 2018), and some studies suggest that declining IFN-γ levels in the IGRA may correlate with reduced bacterial load (Pathan et al., 2001; Carrara et al., 2004; Lalvani, 2004), accumulated evidence over decades has generally questioned its reliability for treatment monitoring. Systematic reviews and meta-analyses have consistently indicated that tracking changes in IGRAs (e.g., using QFT-GIT and T-SPOT.TB) provides limited value for assessing therapeutic efficacy (Pourakbari et al., 2020; Wang et al., 2025).

To address uncertainties regarding IGRA longitudinal variability and IFN-γ response determinants, we aimed to conduct a retrospective analysis of single-center data spanning the past decade. This investigation quantitatively analyzes long-term IFN-γ dynamic changes while employing controlled comparisons to evaluate anti-TB treatment effects. Our findings may offer crucial insights into long-term post-TB infection immune dynamics, assist clinicians in interpreting IGRA results during extended follow-up, and ultimately support refined diagnostic strategies, effective treatment monitoring, and optimized TB management.

## 2 Methods

### 2.1 Study population and methods

This retrospective cohort study utilized clinical data obtained from Shanghai Pulmonary Hospital (Shanghai, China), a tertiary specialized hospital and a national referral center for respiratory diseases. The data were collected between 2013 and 2024, with a total of 90,404 hospitalized patients included. From this initial cohort, 491 eligible patients repeatedly presenting with suspected TB were identified, all of whom had undergone two QuantiFERON-TB Gold (QFT) tests with an inter-test interval of more than 1 month. The first test at the visit for suspected TB was defined as the baseline, and the subsequent test was considered the follow-up. Samples collected within a shorter interval were excluded due to a significantly higher rate of discordant results observed in short-term retesting (21.5% [15.0–28.7%] for intervals ≤1 month vs. longer intervals; P = 0.01) (Wang et al., 2025).

For the enrolled patients, we systematically collected their basic information (including age and sex), the values and timing of two QFT tests, clinical diagnosis data at the time of testing (covering primary diagnosis, TB activity, and extrapulmonary TB status), comorbidities (such as diabetes, coronary heart disease, and hypertension), and anti-TB treatment history and duration. The study was approved by the Medical Ethics Committee of Shanghai Pulmonary Hospital (No.: K25-769) and strictly adhered to the principles of the Declaration of Helsinki. Written informed consent was obtained from all participants.

### 2.2 Diagnostic classification

Participants were classified into three categories based on baseline diagnosis: a TB group, comprising patients with microbiologically or clinically confirmed TB at baseline; a healed TB group, consisting of individuals with radiographic evidence of healed TB, such as fibrotic changes or calcifications, in the absence of clinical activity; and a non-TB group, which comprised patients in whom suspected TB was ruled out after consultation and who were primarily diagnosed with other conditions, including malignancies, nontuberculous mycobacterial infections, and inflammatory disorders.

**Figure 1:**
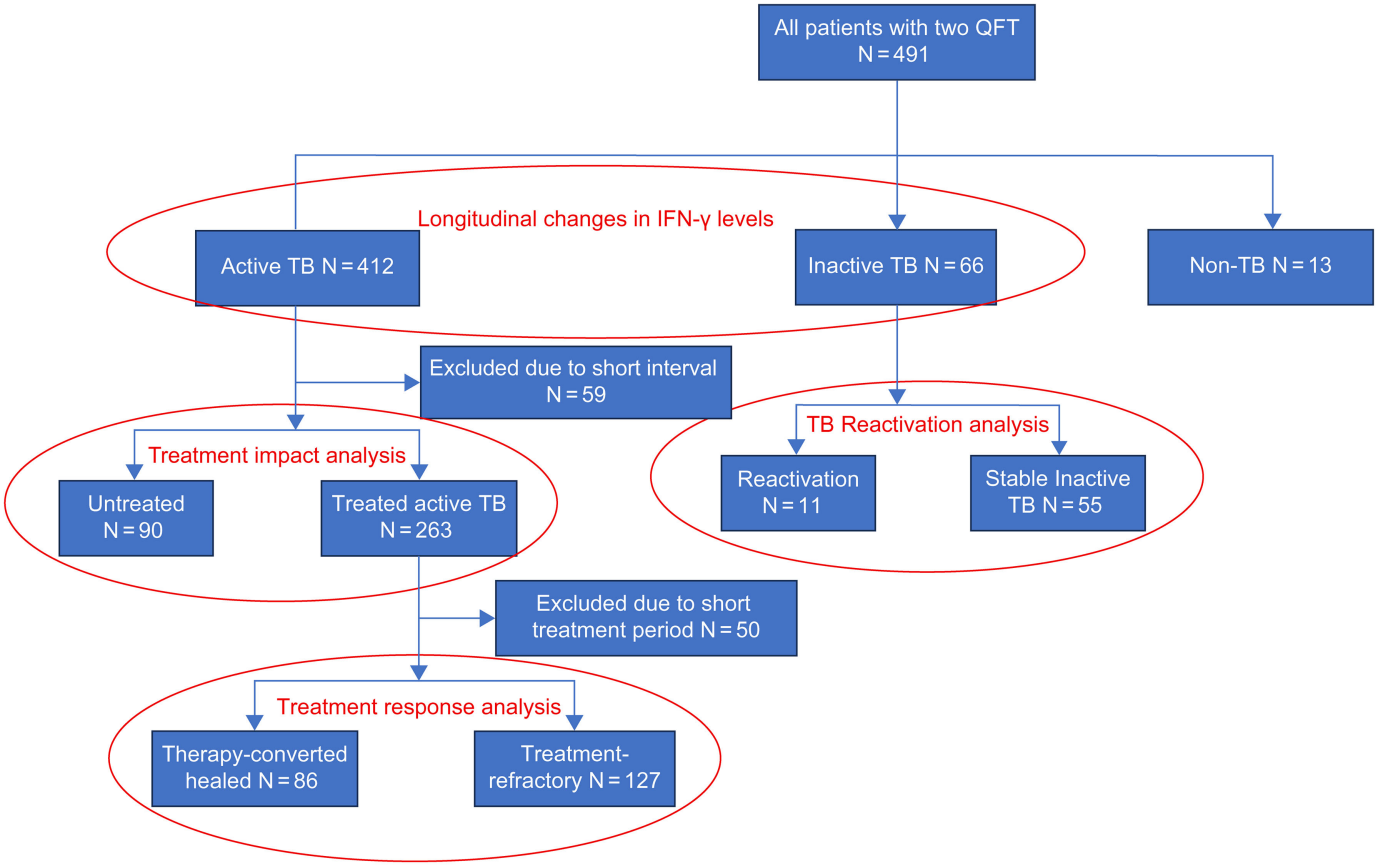
Flowchart of tuberculosis classification and therapeutic stratification protocol based on serial QuantiFERON-TB Gold testing. TB, tuberculosis.

To evaluate the impact of treatment and disease outcomes, stratified analyses were conducted according to the following protocols. (1) In the treatment impact analysis, patients with TB were divided into two subgroups: an untreated TB subgroup, including those who received no antitubercular therapy over a testing interval of at least 2 months; and a treated TB subgroup, comprising patients who had completed a minimum of 2 months of antitubercular therapy. Cases with a testing interval of <2 months were excluded due to insufficient treatment duration. (2) For the treatment response analysis, patients with TB who had undergone at least 6 months of antitubercular therapy were further stratified into a therapy-converted healed subgroup, defined by radiographic confirmation of lesion resolution, and a treatment-refractory subgroup, which included cases of chronic active or recurrent TB with persistent disease. Patients with <6 months of treatment were excluded, as their treatment response was indeterminate. (3) In the reactivation analysis, the healed TB group was subdivided into a stable healed TB subgroup, comprising patients who maintained healed status throughout the observation period, and a reactivation TB subgroup, which included those who developed recurrent TB during follow-up. The complete study algorithm is summarized in **Figure 1**.

### 2.3 QFT assay

The QuantiFERON-TB assay was performed according to the manufacturer’s protocols (QuantiFERON-TB Gold Plus or QuantiFERON-TB Gold In-Tube; Qiagen, Hilden, Germany). The test system included a negative control (nil tube), a positive control (mitogen tube), and for QFT-Plus, antigen tubes TB1 and TB2; for QFT-Gold In-Tube, a single antigen tube containing ESAT-6, CFP-10, and TB7.7 peptides was used. After 16–24 hours of incubation at 37 °C, plasma IFN-γ concentrations were measured. The quantitative result for each antigen tube was calculated as IFN-γ (antigen tube) minus IFN-γ (nil tube). For patients tested with QFT-Plus, results from the TB1 tube were used as QFT-Gold-equivalent values, given the high antigenic agreement.

### 2.4 Statistical analysis

Scatter plots with moving average smoothing were employed to visualize the temporal trends in the magnitude of changes in IFN-γ levels. Additionally, linear regression analysis was conducted to quantify these associations. Subsequent analyses were stratified according to time intervals (1–2, 3–5, 6–11, 12–23, 24–35, 36–47, and ≥48 months; dense in the early stages and sparse in the later stages). Line graphs were generated to depict longitudinal IFN-γ dynamics, and Wilcoxon signed-rank tests were used to evaluate differences within paired samples across intervals. Between-group mean comparisons were performed for each interval, and temporal changes in positivity rates were documented.

For categorical variables (e.g., treatment status, treatment response, diabetes status), an integrated analytical approach was adopted. This approach comprised: (1) visualization using scatter plots with moving average trends; (2) quantitative statistical analysis, including between-group mean comparisons and Wilcoxon signed-rank tests for paired samples; and (3) assessment of the discriminatory power of ΔIFN-γ levels using receiver operating characteristic (ROC) curve analysis.

For annual slope calculation and analysis, the annual slope of ΔIFN-γ levels was calculated for each patient to evaluate the declining trend over time. The distribution of annual slopes was assessed using measures of central tendency (mean and median), dispersion (standard deviation [SD] and interquartile range [IQR]), and tail proportions. Normality was examined by visual inspection of the distributions. Comparisons across multiple follow-up time intervals were performed using the Kruskal–Wallis test. Between-group differences (treated vs. untreated and therapy-converted healed vs. treatment-refractory) were evaluated using the Mann–Whitney U test. A two-sided P < 0.05 was considered statistically significant.

Linear regression analyses were conducted to evaluate associations between ΔIFN-γ levels and covariates including sex, age, diabetes status, extrapulmonary TB, treatment status, treatment impact, treatment response, and testing interval. Variables with P < 0.10 in univariate analysis were entered into a multivariable regression model. Variables that showed significant association (P < 0.05) in the final model, such as diabetes, were further analyzed through subgroup analysis consistent with the approach used for categorical variables. Linear mixed-effects models with a random intercept for patient ID were used to analyze longitudinal QFT changes, including the treatment × time interaction and covariates as fixed effects. Two models were constructed using different treatment definitions (treatment impact vs. treatment response). The intraclass correlation coefficients were calculated. Statistical significance was set at P < 0.05.

All statistical analyses were performed using Python (v3.9; packages: pandas, scipy, statsmodels, matplotlib, seaborn) and SPSS (v26, IBM Corp., Armonk, NY). Data visualization was primarily implemented with the Matplotlib and Seaborn libraries.

## 3 Results

### 3.1 Baseline characteristics of the study participants

A total of 491 participants were included in this study and categorized into three groups: TB (n = 412), healed TB (n = 66), and a non-TB control group (n = 13). The baseline demographic and clinical characteristics of the participants are summarized in **Table 1**. The mean age was highest in the non-TB group (63 ± 9 years), with values of 50 ± 18 years in the TB group and 53 ± 15 years in the healed TB group. Men predominated across all groups, with the highest proportion in the non-TB group (77%). Among patients with TB, pulmonary TB was more common in the healed TB group (83%), while extrapulmonary TB was more frequent in the TB group (37%). The prevalence of comorbid diseases, including diabetes mellitus, coronary heart disease, and hypertension, was generally comparable among the groups, with hypertension being slightly more common in the non-TB group (23%).

**Table 1:** Baseline demographic and clinical characteristics.

Index	TB group *N = 412*	Healed TB group *N = 66*	Non-TB group *N = 13*
Age, years	50 ± 18	53 ± 15	63 ± 9
Sex, male	284 (69)	40 (61)	10 (77)
PTB only (%)	259 (63)	55 (83)	0 (0)
EPTB (%)	153 (37)	11 (17)	0 (0)
Concomitant diseases (%)			
Diabetes mellitus	62 (15)	10 (15)	2 (15)
Coronary heart disease	19 (5)	5 (8)	1 (8)
Hypertension	71 (17)	10 (15)	3 (23)

Data are presented as mean ± standard deviation (SD) or n of patients (%). *N* represents the number of enrolled patients. TB, tuberculosis; PTB, pulmonary tuberculosis; EPTB, extrapulmonary tuberculosis.

### 3.2 Longitudinal changes in QFT IFN-γ levels and positivity rates

**Figure 2:**
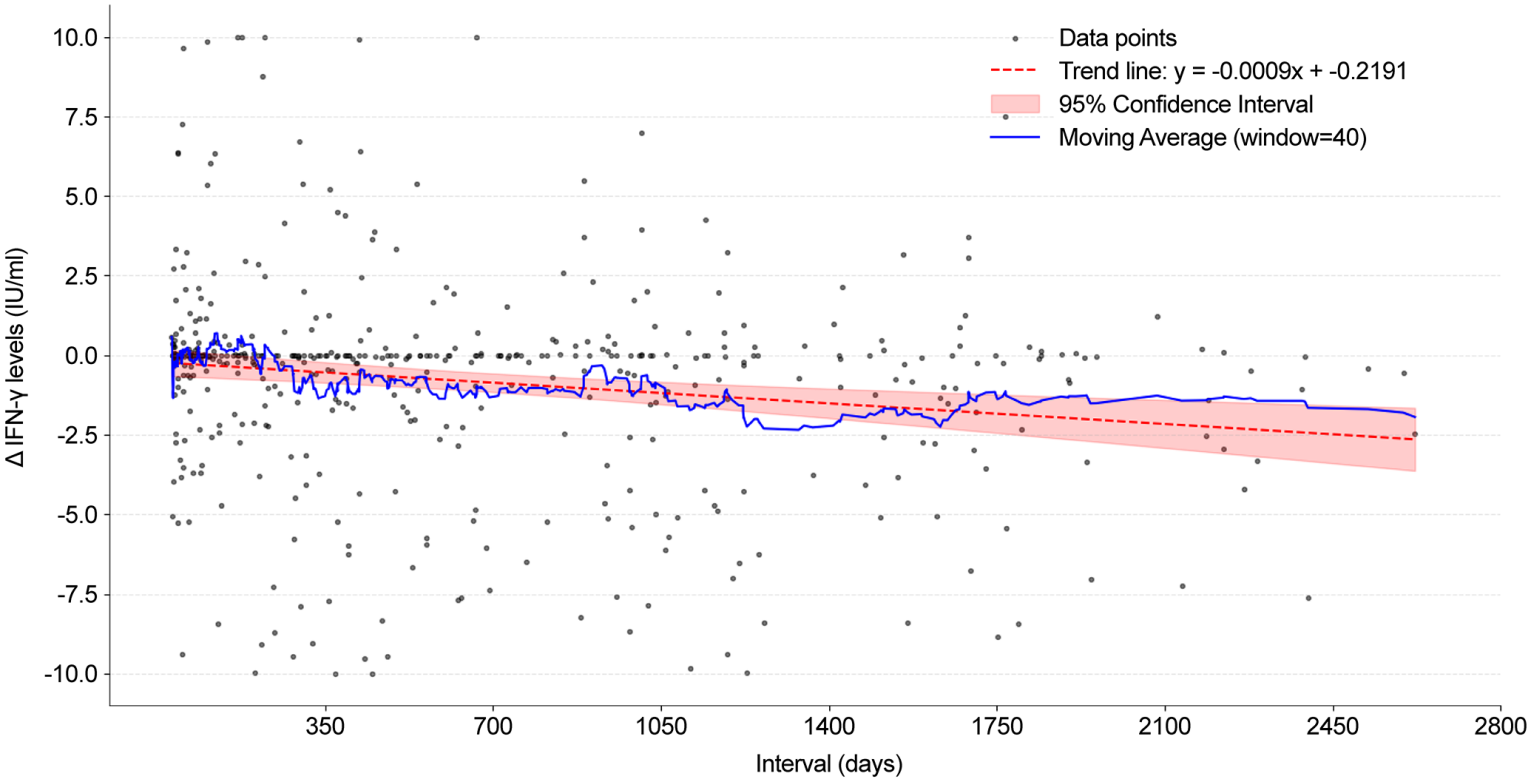
Longitudinal ΔIFN-γ changes over time. Scatter plot showing ΔIFN-γ levels over time in eligible participants who completed two QFT tests (n = 491), with linear trendline, 95% confidence interval, and moving average. The black circles (●) represent individual data points showing the changes in IFN-γ levels (ΔIFN-γ) at corresponding time intervals (days). The red dashed line depicts the linear trendline (y = −0.0009x − 0.2191). The shaded area around the trendline represents the 95% confidence interval for the linear regression fit. The blue solid line represents the moving average (window = 40). QFT, QuantiFERON-TB Gold; IFN-γ, interferon-gamma.

A statistically significant but weak negative linear trend between ΔIFN-γ levels and time intervals was observed using scatter plot visualization and simple linear regression (ordinary least squares) analysis (r = −0.1736, P = 0.0001). Although substantial heterogeneity was observed across the time range, the overall distribution of ΔIFN-γ values demonstrated a downward shift with increasing interval duration, progressively dispersing toward the negative side of the y-axis (**Figure 2**). The fitted trendline (y = −0.0009x − 0.2191) indicated a gradual decline in IFN-γ levels over time, with an estimated rate of −0.3285 IU/year. Given typical IFN-γ levels of approximately 4 IU/mL, this absolute decrease corresponds to a relative decline of roughly 10% per year. The model goodness-of-fit, assessed by a root mean square error (RMSE) of 3.2651 IU/mL and a relative RMSE (RRMSE) of 16.33%, indicated an acceptable (10% ≤ RRMSE < 20%) but not excellent fit (RRMSE < 10%), with substantial predictive uncertainty. The low R^2^ value (0.0301) confirmed that time intervals explained only a minor fraction of the variability in ΔIFN-γ levels.

**Figure 3:**
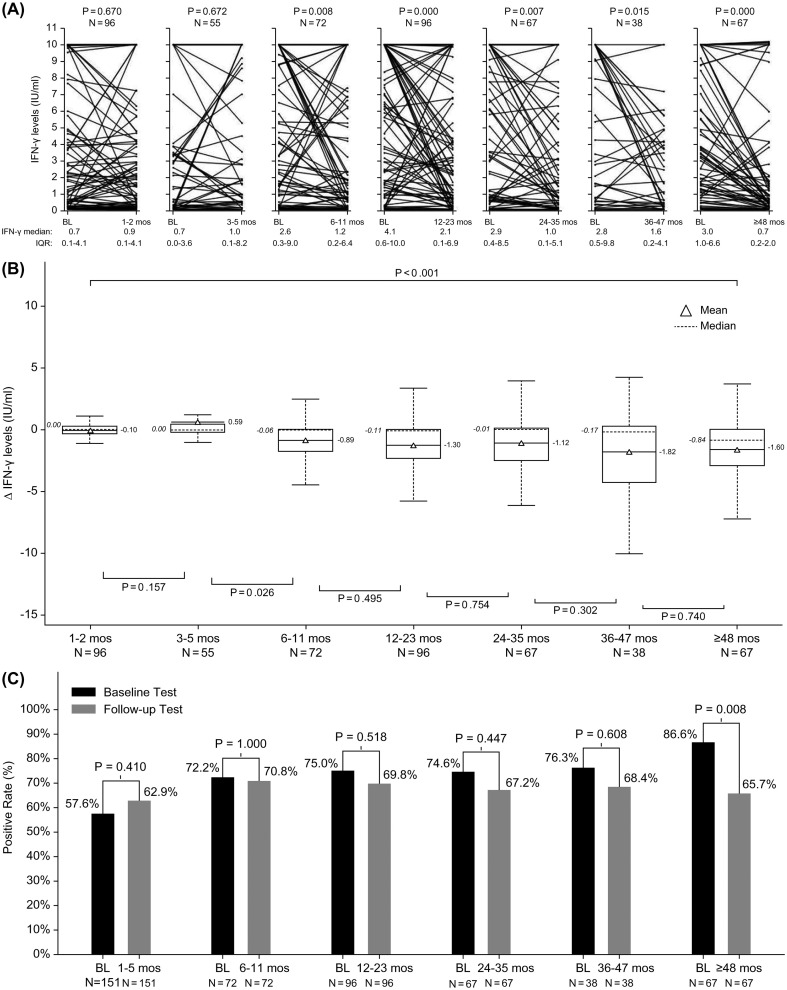
Quantitative longitudinal analysis of IFN-γ dynamics and QFT positivity rates by time interval. **(A)** Temporal evolution of IFN-γ levels across different time intervals in eligible participants who completed two QFT tests (n = 491). Statistical significance is determined by the Wilcoxon signed-rank test, with a significance level set at P < 0.05. BL, baseline; mos, months. **(B)** Distribution of the change in IFN-γ levels (ΔIFN-γ, IU/mL) across different time intervals (n = 491). Box plots depict the median (dashed line inside the box), interquartile range (box boundaries), and the full data range within 1.5 × IQR (whiskers). Triangles (▲) represent mean values. Numerical values for the median and mean are displayed on the left and right sides of each box plot, respectively. The top horizontal line indicates the P-value for the overall between-group comparison (one-way analysis of variance). The bottom horizontal lines indicate P-values for pairwise comparisons between adjacent time points (two-sample t-test), with statistical significance set at P < 0.05. **(C)** QFT positivity rates at baseline and during follow-up for each corresponding time interval. IQR, interquartile range; QFT, QuantiFERON-TB Gold; IFN-γ, interferon-gamma.

Stratified analysis of QFT-Plus data revealed distinct temporal dynamics in IFN-γ levels. No significant changes were observed within short intervals (<6 months; 0–2 months: P = 0.670; 3–5 months: P = 0.672). In contrast, intervals of ≥6 months exhibited progressive and significant declines (e.g., 6–11 months: P = 0.008; 12–23 months: P < 0.001), which were accompanied by a narrowing of the interquartile ranges (**Figure 3A**). Analysis of ΔIFN-γ levels confirmed significant variations across the time intervals (one-way analysis of variance, P < 0.001). *Post-hoc* pairwise comparisons indicated that a significant decrease occurred between the 3–5- and 6–11-month groups (P = 0.026). Furthermore, Levene’s test demonstrated a significant widening of the interquartile ranges over time (P = 0.029), indicating increasing heterogeneity in the IFN-γ response with longer intervals (**Figure 3B**). Conversely, the decline in QFT positivity rates occurred more gradually than the reduction in IFN-γ levels. A significant decrease in the positivity rate (from 0.82 to 0.60; P = 0.008) was only observed after an interval exceeding 4 years, with no significant differences detected at any shorter follow-up time points (**Table 2**; **Figure 3C**).

**Table 2:** Longitudinal changes in QFT positivity rates stratified by follow-up time intervals.

Time period (months)	Participants, n	Positivity rates at baseline (%)	Positivity rates at follow-up test (%)	Difference in positivity rates between baseline and follow-up test	P-value
1–5	151	57.6	62.9	−5.3	0.410
6–11	72	72.2	70.8	1.4	1.000
12–23	96	75.0	69.8	5.2	0.518
24–35	67	74.6	67.2	7.4	0.447
36–47	38	76.3	68.4	7.9	0.608
≥48	67	86.6	65.7	20.9	**0.008**

Bold P-values indicate statistical significance (P < 0.05). QFT, QuantiFERON-TB Gold.

### 3.3 Impact of disease status on longitudinal changes in QFT IFN-γ levels

Longitudinal dynamics between patients with TB (n = 412) and healed TB (n = 66) were compared using moving average smoothing (window size = 40). The analysis demonstrated that the healed TB group consistently maintained higher IFN-γ levels throughout the observation period (**Supplementary Figure 1**), with a mean difference of 0.75 IU/mL (SD = 0.43) between the smoothed trajectories. This difference was highly significant (t = 17.52, P < 0.0001), consistent with a more rapid decline in IFN-γ levels in the TB group.

### 3.4 Impact of anti-TB treatment on longitudinal changes in QFT IFN-γ levels

**Figure 4:**
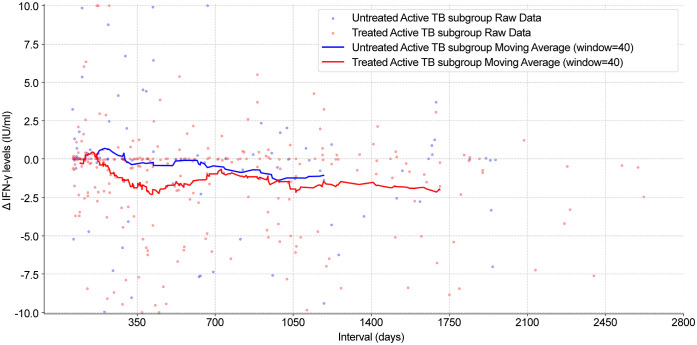
Temporal trends of ΔIFN-γ levels between treated and untreated groups. Scatter plots and moving average curves (window size = 40) illustrate temporal changes in ΔIFN-γ levels in treated and untreated groups. IFN-γ, interferon-gamma.

Scatter plots of ΔIFN-γ levels against time intervals overlaid with moving average trendlines (window size = 40) revealed distinct longitudinal patterns between treated (N = 263) and untreated (N = 90) patients, characterized by a clear separation of the smoothed trajectories. Statistical analysis quantified a mean difference (treated − untreated) of −0.8926 IU/mL (SD = 0.5463), indicating systematically lower ΔIFN-γ levels in the treated group throughout the observation period. A paired t-test confirmed that this difference was significant (t = −16.2582, P < 0.001), demonstrating a lower temporal trend of ΔIFN-γ levels in the treated group (**Figure 4**).

**Figure 5:**
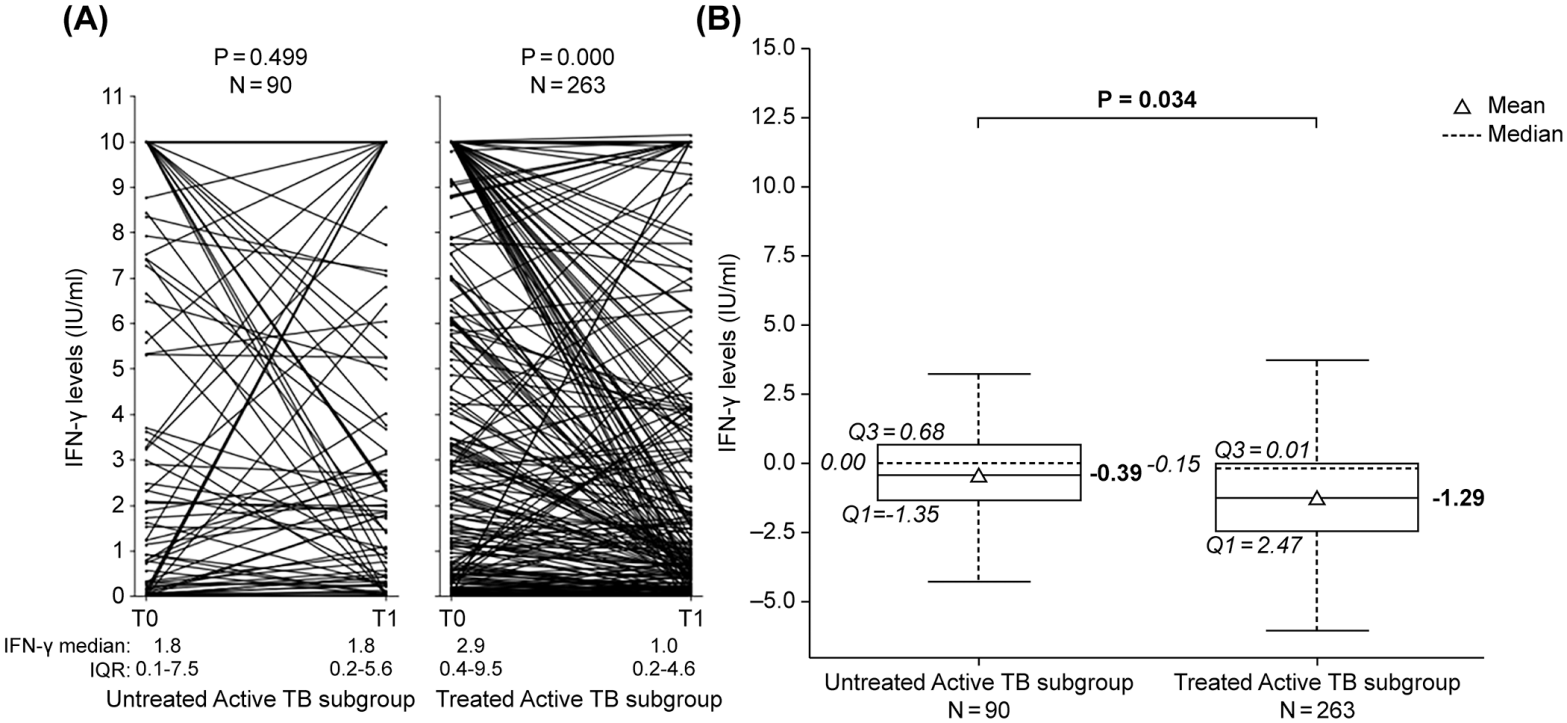
Quantitative analysis of IFN-γ levels in untreated and treated TB subgroups. **(A)** Paired comparison of quantitative IFN-γ levels by Wilcoxon signed-rank test. **(B)** Comparison of the change in quantitative IFN-γ levels (ΔIFN-γ) between subgroups. TB, tuberculosis; IFN-γ, interferon-gamma.

A paired analysis of quantitative IFN-γ levels before and after anti-TB treatment using the Wilcoxon signed-rank test revealed a marked decline in the treated group but not in the untreated group. In the untreated TB subgroup (N = 90), IFN-γ levels remained stable between T0 and T1 (P = 0.499; median: 1.8 IU/mL at both time points). In contrast, a significant decrease was observed in the treated subgroup (N = 263, P < 0.001; median level declined from 2.9 IU/mL at T0 to 1.0 IU/mL at T1) (**Figure 5A**). Consistently, the reduction in IFN-γ levels (ΔIFN-γ) was significantly greater in the treated subgroup (P = 0.034, **Figure 5B**). However, considerable overlap in ΔIFN-γ levels distribution was observed between subgroups, with IQRs of (−1.35, 0.68) for untreated and (−2.47, 0.01) for treated patients. Accordingly, ROC analysis showed limited discriminative ability [area under the curve (AUC) = 0.582, **Supplementary Figure 2**].

### 3.5 Impact of treatment response on longitudinal changes in QFT IFN-γ levels

**Figure 6:**
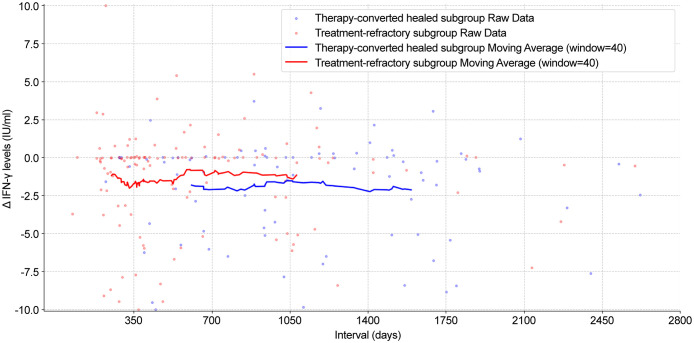
Temporal trends of ΔIFN-γ levels between therapy-converted and treatment-refractory subgroups. Scatter plots and moving average curves (window size = 40) illustrate temporal changes in ΔIFN-γ levels in therapy-converted and treatment-refractory subgroups. IFN-γ, interferon-gamma.

**Figure 7:**
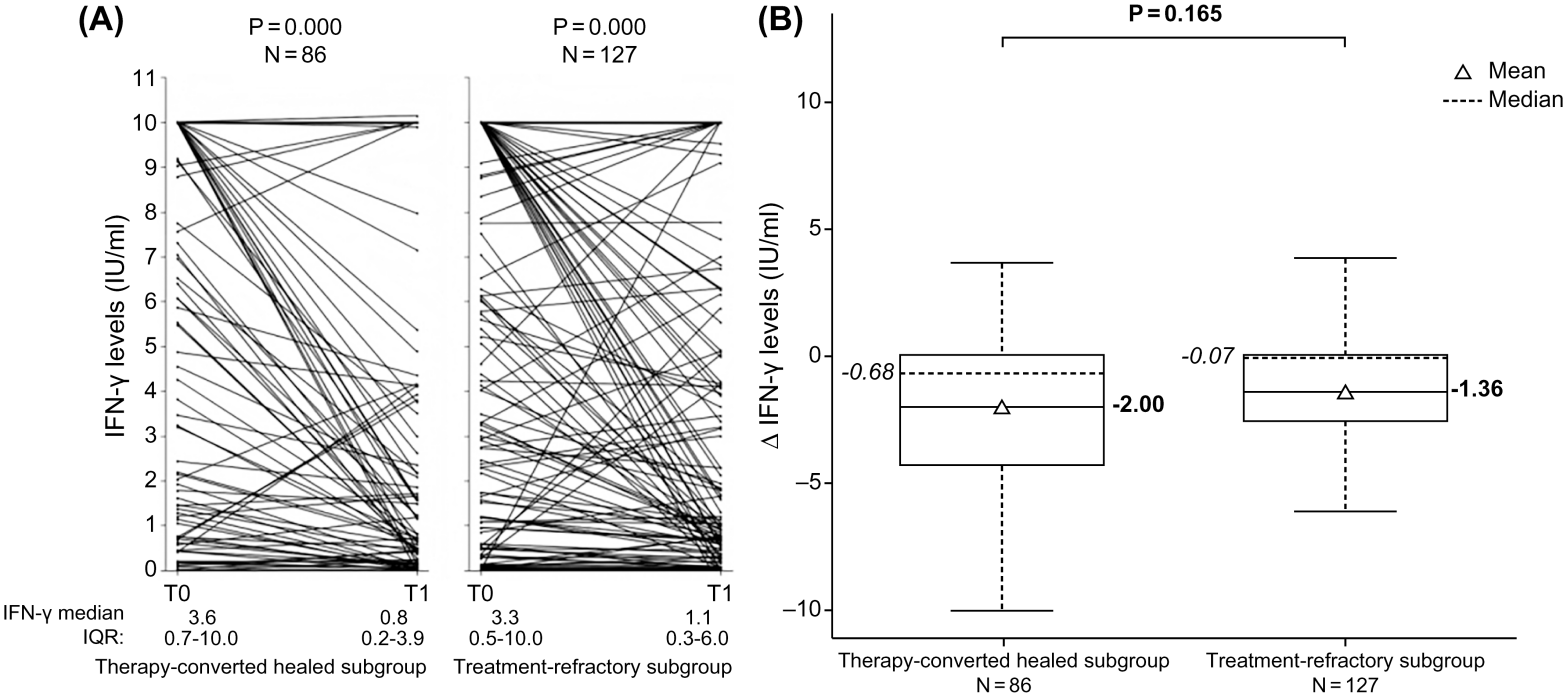
Quantitative analysis of IFN-γ levels in therapy-converted and treatment-refractory TB subgroups. **(A)** Paired comparison of quantitative IFN-γ levels by Wilcoxon signed-rank test. **(B)** Comparison of quantitative ΔIFN-γ levels between therapy-converted and treatment-refractory subgroups. TB, tuberculosis; IFN-γ, interferon-gamma.

Scatterplot visualization with moving average smoothing (window size = 40) revealed divergent temporal dynamics in ΔIFN-γ levels between the therapy-converted healed (N = 86) and treatment-refractory (N = 127) subgroups (**Figure 6**). Throughout the observation period, the moving average curve was consistently lower in the therapy-converted healed subgroup than in the refractory subgroup. The mean difference between the smoothed trajectories was 0.8175 IU/mL (SD = 0.2977), with the small SD indicating a stable separation. The difference was significant (t = 27.32, P < 0.0001). However, despite this apparent divergence in temporal patterns of ΔIFN-γ, the overall comparison revealed that the difference between subgroups was not statistically significant. Although subsequent application of the Wilcoxon signed-rank test revealed a significant post-treatment decrease in quantitative IFN-γ levels in both subgroups (therapy-converted healed: median T0 3.6 IU/mL vs. T1 0.8 IU/mL, P < 0.001; treatment-refractory: median T0 3.3 IU/mL vs. T1 1.1 IU/mL, P < 0.001; **Figure 7A**), the overall difference in ΔIFN-γ levels between the two subgroups was not significant (P = 0.165, **Figure 7B**), with considerable within-group heterogeneity and overlapping distributions. ROC analysis further confirmed the limited discriminative ability of ΔIFN-γ (AUC = 0.551, **Supplementary Figure 3**), suggesting that although influenced by treatment response, it is not a reliable distinguishing marker.

### 3.6 Annual slope of IFN-γ changes: distribution, temporal patterns, and treatment-related differences

We further calculated the annual slope of IFN-γ changes to evaluate the declining trend over time. Overall, the distribution of annual IFN-γ slope was approximately normal and symmetric (**Supplementary Figure 4A**), with a mean of −0.48 ± 9.44 and a median of −0.04. The majority of data fell between −1.34 and 0.08 (IQR = 1.43), with a small proportion of extreme values on both tails (2.4% below −20 and 2.0% above 20). Stratification by follow-up time intervals (**Supplementary Figure 4B**) showed that, except for the 3–5-month group exhibiting a positive mean slope (1.71/year) driven by two extreme values, the mean slopes of the other intervals ranged from −1.2 to −0.3 per year, with no significant overall difference among groups (Kruskal–Wallis test, H = 7.770, P = 0.255). Among patients with active TB (**Supplementary Figure 4C**), the treated group had a median annual IFN-γ slope of −0.093, while the untreated group had a median of 0.000, with a statistically significant difference (Mann–Whitney U = 14213.5, P = 0.004). However, substantial overlap in IQRs (untreated: −0.596 to 0.695; treated: −1.643 to 0.004) and large SDs indicated high within-group variability. Further comparison between treatment response subgroups among treated patients (**Supplementary Figure 4D**) revealed no statistically significant differences in the means or medians of annual IFN-γ slopes between the therapy-converted healed subgroup and the treatment-refractory subgroup (Mann–Whitney U test, P = 0.706).

### 3.7 Factors associated with longitudinal changes in QFT IFN-γ levels

In both univariable and multivariable analyses, diabetes (coefficient: −1.082, P = 0.040), treatment impact (coefficient: −1.015, P = 0.017), and testing interval (coefficient: −0.001, P = 0.006) emerged as independent factors significantly associated with changes in IFN-γ levels. The variance inflation factors for all these variables were below 1.2. The complete results are summarized in **Table 3**, and diabetes was newly identified as a significant factor associated with longitudinal QFT IFN-γ dynamics.

**Table 3:** Univariable and multivariable linear regression analyses of longitudinal changes in IFN-γ levels.

Variable	Univariable regression					Multivariable regression	
	Coefficient (95% CI)	P-value	Coefficient β	R^2^	Coefficient (95% CI)	P-value	VIF
Sex	−0.443 (−1.090; 0.205)	0.180	−0.061	0.004	–	–	–
Age (years)	−0.016 (−0.032; 0.001)	0.071	−0.082	0.007	−0.013 (−0.033; 0.007)	0.213	1.071
Diabetes	−1.182 (−2.021; −0.343)	0.006	−0.124	0.015	−1.082 (−2.116; −0.049)	0.040	1.106
Extrapulmonary TB	0.215 (−0.431; 0.862)	0.513	0.030	0.001	–	–	–
Treatment impact	−0.889 (−1.729; −0.048)	0.038	−0.110	0.012	−1.015 (−1.848; −0.183)	0.017	1.020
Treatment response	−0.187 (−1.000; 0.626)	0.651	−0.028	0.001	–	–	–
Interval (days)	−0.001 (−0.001; 0.000)	0.000	−0.166	0.028	−0.001 (−0.001; 0.000)	0.006	1.013

A detailed analysis of temporal trends (diabetic vs. non-diabetic) was conducted for the candidate factor, diabetes, to elucidate its impact on longitudinal IFN-γ dynamics. Longitudinal visualization with moving average smoothing confirmed distinct trajectories between the two groups, showing consistently lower levels in the diabetic subgroup (mean difference: −1.0981 IU/mL, SD = 0.5047; t = −21.6483, P < 0.0001) (**Supplementary Figure 5**). The Wilcoxon signed-rank test revealed a significant decline in IFN-γ levels in both the non-diabetic group (N = 417, P < 0.001; median: 2.1 to 1.1 IU/mL) and the diabetic group (N = 74, P < 0.001; median: 3.1 to 1.1 IU/mL) (**Supplementary Figure 6A**). Comparative analysis indicated a more pronounced reduction in ΔIFN-γ in the diabetic group (mean: −1.85 vs. −0.67 IU/mL, P = 0.006), although substantial within-group heterogeneity was observed (|coefficient of variation| = 1.74 vs. 5.08) (**Supplementary Figure 6B**). Collectively, these results demonstrate that diabetes significantly modulates both the extent and temporal dynamics of IFN-γ decline.

### 3.8 Linear mixed-effects model results stratified by treatment definition

**Supplementary Table 1** compares the linear mixed-effects model results for factors associated with QFT changes using two different treatment definitions (treatment impact vs. treatment response). Model 1 (treatment impact) showed a significant treatment × time interaction (coefficient = −0.889, P = 0.038), indicating a significant treatment effect. In contrast, Model 2 (treatment response) did not show a significant interaction (coefficient = −0.641, P = 0.167), suggesting no significant treatment effect. Age was significantly negatively associated with QFT values in both models (both P < 0.001). Sex (P = 0.039) and extrapulmonary TB (P = 0.019) were significant covariates in Model 1 but not in Model 2. The intraclass correlation coefficient values were 0.563 and 0.606, respectively, supporting the use of mixed-effects models.

## 4 Discussion

Longitudinal quantitative changes within the same individuals provided greater precision than binary positive/negative classification, while the inclusion of untreated and successfully treated cohorts as controls enhanced causal inference by reducing confounding variables and selection bias. Together, these approaches not only enabled a comprehensive characterization of the QFT trajectory following Mtb infection and identification of its modulating factors, but also provided direct evidence that IFN-γ levels—as measured by IGRA—and the immune reactivity they represent were not stable over the long term but rather exhibited a natural waning trend across patient populations. This finding is consistent with the approximately 10% annual reversal rate of TST reactivity reported in the 1948 Prophit Survey (Daniels et al., 1948). Our analysis further revealed that intervals exceeding 6 months were sufficient to detect statistically significant declines in IFN-γ levels, a trend that persisted throughout the subsequent 4-year observation period. The magnitude of this decline can be roughly estimated using the linear regression equation derived from our cohort (ΔIFN-γ = −0.0009 × time in days − 0.2191), which corresponds to an estimated rate of decline of approximately −0.33 IU/mL per year. This downward trend was supported by a significant negative correlation confirmed via Pearson correlation analysis. These findings challenge the conventional dogma of persistent immunoreactivity and necessitate a critical re-evaluation and refinement of clinical interpretation of serial IGRA results, epidemiological models of Mtb infection (such as those estimating the annual risk of infection or latency), and public health strategies reliant on the assumption of stable immunoreactivity—including the notion that a single positive test result implies lifelong risk.

Although we have obtained direct evidence of a natural waning in IFN-γ levels, this pattern cannot yet be leveraged to infer disease course in individual patients, primarily due to substantial heterogeneity in the data. This is evidenced by the wide SDs and IQRs (e.g., IQRs spanning several IU/mL across all intervals) and a model RMSE of 3.265 IU/mL, which is a crucial finding. The predictive power of the regression model (ΔIFN-γ = −0.0009 × time in days − 0.2191) is limited, with a low R^2^ value (0.0301), indicating that time explains only a minor fraction of the variability in individual IFN-γ dynamics. Consequently, the considerable predictive uncertainty, reflected by an RRMSE of 16.33%, translates to a potential error of approximately ±3.27 IU/mL for any individual prediction. This error magnitude is substantial when compared to the estimated annual mean decline of −0.33 IU/mL, rendering the formula unsuitable for predicting precise changes at the individual level. Nonetheless, the model retains value for describing the central tendency at the population level. According to the law of large numbers, the aggregation of data from a large cohort mitigates the effect of individual variability, allowing the model to provide a reasonable estimate of the average waning trend expected in a similar population over time.

Our data revealed that the decline in IFN-γ levels measured using the IGRA was very subtle and remained undetectable through changes in QFT positivity rates. Building upon the observed dynamic patterns of IFN-γ levels, our findings further indicated that, although quantitative QFT values declined significantly over time, the reduction in test positivity (using the 0.35 IU/mL cutoff) did not reach statistical significance until the 4-year follow-up, which can be attributed to two interrelated factors: the inherently slow rate of IFN-γ waning and substantially elevated baseline values above the diagnostic threshold. For instance, patients with TB exhibited a mean initial QFT value of approximately 4.0 IU/mL, and given the projected population-level waning rate of 0.33 IU/mL per year derived from our regression model, it would require more than a decade for the average value to approach the cutoff—an observation consistent with the findings of the classic Prophit Survey (Daniels et al., 1948), which reported an annual reversal rate of TST reactivity of approximately 10%. In contrast, for patients with TB with low initial IFN-γ levels, the time to lose IFNg+ status could be less than 5 years. This finding may partly explain the pervasive notion that “a positive TST/IGRA persists lifelong.”

Furthermore, our results align with studies demonstrating an inverse relationship between reversion probability and baseline IFN-γ levels, as corroborated by Jonsson et al. (2017), who reported a 54.7% reversion rate within 3–12 weeks among individuals with baseline IFN-γ levels of 0.35–0.99 IU/mL. These results are similar to those observed by Pérez-Recio et al. (2023), where sustained reversion occurred almost exclusively in this low-range category (4 of 96 reverters), and are further supported by a study of Indian household contacts documenting a 12-month reversion rate of 20% for those with baseline levels of 0.35–0.70 IU/mL versus an overall rate of only 6.7% (Pai et al., 2009). The study by Schmidt et al. demonstrated that Bacille Calmette-Guérin revaccination did not significantly reduce the incidence of “sustained infection,” which is defined as an initial IGRA conversion without subsequent reversion. This is evidenced by the non-significant difference in IGRA reversion rates between the Bacille Calmette-Guérin vaccine and placebo groups (44% vs. 42%) (Schmidt et al., 2025). Collectively, these findings underscore that the slow natural decline of IGRA values is a fundamental driver of low reversion rates at the population level, suggesting that future longitudinal studies should prioritize the analysis of continuous quantitative changes in IFN-γ rather than relying solely on binary conversion metrics to more accurately elucidate the nuanced immunodynamics of Mtb infection.

Our data further suggest that the patterns of changes in IFN-γ levels may differ across stages following TB. Stratified subgroup analysis by diagnostic status, employing moving average smoothing, revealed that IFN-γ levels exhibited a declining trend over time in both TB and healed TB groups, although the decrease in the healed TB group did not reach statistical significance. The moving average curve for the TB group remained consistently lower than that of the healed TB group throughout the observation period, with a mean difference of 0.75 IU/mL (SD = 0.431), suggesting a more rapid decline in IGRA responses among patients with TB. This phenomenon may be attributable to the rapid clearance of Mtb antigens following the initiation of anti-TB therapy, which subsequently reduces the persistent antigenic stimulation required to maintain T-cell reactivity. Further examination of the moving average curve morphology indicated that the IFN-γ dynamics in TB patients followed a phasic pattern: a minor initial increase within the first 200 days followed by a sharp decline between 200 and 300 days and ultimately transitioning into a fluctuating plateau phase after 300 days. In contrast, the healed TB group (data available from approximately 400 days onward) demonstrated only a slight initial decrease before entering a similar fluctuating plateau. This temporal pattern closely mirrors the theoretical dynamics of mycobacterial antigen load, namely initial accumulation, rapid clearance (potentially treatment-induced), and eventual stabilization. Although these observations remain descriptive and lack further statistical confirmation, they provide novel insights into the dynamic changes in immune responses during TB progression. Furthermore, they highlight the need for future longitudinal studies with higher sampling frequencies to formally test the potential link between immune response dynamics and bacterial antigen kinetics.

We posit that while monitoring treatment efficacy via IFN-γ levels is theoretically feasible, it is not yet technically operable. Accumulated evidence over decades has generally questioned its reliability for treatment monitoring. Dale et al. argued that this is primarily because the immune reactivity reflects the magnitude of established immune memory from past antigen exposure rather than real-time diagnosis of viable bacteria (Dale et al., 2024), and treatment does not significantly increase IGRA reversion rates (Pourakbari et al., 2020). Prior studies have often faced methodological constraints, such as reliance on qualitative conversion/reversion outcomes rather than continuous quantitative analysis, and a general lack of well-controlled comparative designs.

Our study sought to address some of these limitations through improved design and data analysis. We provide direct evidence of the effects of treatment and treatment response on IGRA-measured IFN-γ levels: We observed that patients receiving anti-TB treatment exhibited a more pronounced decline in IFN-γ levels compared to untreated controls; similarly, moving-average-smoothed temporal trends revealed a greater decline in the treatment success group over extended follow-up. However, these statistically significant group-level effects must be sharply distinguished from clinical utility at the individual patient level. Substantial inter-individual variability resulted in extensive data overlap, rendering ΔIFN-γ ineffective for clinically distinguishing treatment status or treatment response. Thus, while our findings confirm a measurable treatment effect at the population level, the excessive inter-individual variability renders this effect far too weak and noisy to serve as a reliable guide for individual clinical decision-making—a conclusion that aligns with the prevailing literature consensus that high individual variability, likely stemming from both assay imprecision and host immune heterogeneity, limits its potential as a standalone biomarker for treatment monitoring (Min, 2025). This underscores the urgency of developing novel high-precision immunoreactivity testing tools. For instance, the novel VIDAS TB-IGRA, benefiting from its fully automated process, significantly reduces indeterminate rates and promises to be an IGRA tool with lower variability and more reliable results (Serge Diagbouga et al., 2025).

Our findings suggest a more complex relationship between IFN-γ production by sensitized T cells in peripheral blood and mycobacterial antigen load than previously recognized. While earlier studies proposed a correlation between IFN-γ levels in QFT assays and the Mtb antigen burden (Day et al., 2011)—explaining the stable IFN-γ levels in patients with untreated TB—we observed a significant decline in IFN-γ levels regardless of treatment response, with only modest differences in the magnitude of decline. This contradicts the theoretical expectation that persistent antigen in treatment-failure cases should maintain elevated IFN-γ responses. We propose two explanatory hypotheses. First, the relationship between antigen load and IFN-γ response may not be linear; a minor reduction in antigen early in treatment could trigger a substantial decline in IFN-γ, which may remain elevated even after antigen clearance, indicating that IFN-γ levels do not accurately reflect dynamic antigen changes. Second, since the IGRA primarily detects sensitized T cells in peripheral blood, its readout is more dependent on systemic immune status than on local disease activity. Drug treatment may rapidly clear blood-borne antigens, leading to a reduction in circulating T-cell responses and QFT values, independent of the actual pathogen clearance at the lesion site. This interpretation is supported by previous reports of significantly higher IGRA false-negative rates in skeletal TB compared to lymphatic TB (55% vs. 14%; Song et al., 2009), suggesting that localized immune responses may not be fully recapitulated in the systemic circulation. Further studies directly examining T-cell responses at lesion sites are warranted to validate these hypotheses.

To investigate the potential factors influencing the long-term trajectory of immunoreactivity variability of QFT in the post-active TB phase, this analysis revealed for the first time that diabetes mellitus was an independent negative predictor of longitudinal IFN-γ decline, suggesting that hyperglycemia or associated immunometabolic disturbances may impair sustained IFN-γ expression. No significant associations were observed for age, sex, or extrapulmonary TB in the adjusted model. Subsequent paired and longitudinal analyses further demonstrated a significantly greater reduction in IFN-γ levels in diabetes patients than in individuals without diabetes, with this difference persisting in time-trend analyses. Patients with diabetes often exhibited higher baseline IFN-γ levels followed by a steeper decline, potentially indicating an initially robust immune response to Mtb infection that is poorly sustained, leading to a more rapid decrease thereafter. While direct evidence linking diabetes to IGRA reversion remains scarce, our study provides preliminary insights into this association. However, despite its statistical significance, diabetes explained only a minimal portion of the observed variance, indicating that the dynamics of IFN-γ responses are predominantly driven by other, unaccounted factors. Future studies with larger cohorts and more frequent sampling are warranted to systematically elucidate the drivers of QFT variability.

This study had some limitations. First, the retrospective, single-center design may have introduced unmeasured confounding factors, and caution is warranted when generalizing the findings. The availability of QFT results at only two time points per patient, without more intensive dynamic monitoring, limited the ability to precisely delineate the continuous trajectory of IFN-γ levels, a limitation largely attributable to the high cost of QFT and the conventional view of lifelong positivity, which make repeated testing uncommon in clinical practice; LTBI patients, being asymptomatic and not requiring hospitalization, lack post-treatment QFT data as well. Although a prospective design with serial testing would be methodologically advantageous, it could raise ethical concerns in this context due to potential delays in treatment initiation. In contrast, our retrospective approach enabled the inclusion of patients with untreated TB as a control group, which is often logistically or ethically challenging in interventional studies. In addition, information on BCG vaccination status was not available for our cohort, as most adult patients could not accurately recall their childhood vaccination history, and this precluded further exploration of its potential influence on longitudinal IFN-γ dynamics via immune modulation. Second, the analysis was primarily based on the standard QFT assay; we could not fully leverage the QFT-Plus system for deeper investigation. Specifically, limited data from the TB2 antigen tube precluded a robust assessment of its potential for improved accuracy or reduced variability in treatment monitoring (Kamada and Amishima, 2017). Furthermore, the study cohort did not include individuals with Mtb infection or pediatric patients, thus the generalizability of our conclusions across different infection statuses and age groups requires further validation.

Nevertheless, this study provided early evidence for a dynamic rather than static immune response during TB treatment, as reflected in the quantitative decline in IFN-γ levels, which may reflect long-term attenuation of or adaptation to mycobacterial antigens. However, future prospective, multi-center studies incorporating dynamic multi-timepoint monitoring and multi-parameter immune assessments, including QFT-Plus, are required to elucidate immune dynamics during TB treatment.

## 5 Conclusions

IFN-γ levels demonstrated a dynamic but highly variable decline over time, confirming attenuation of IGRA reactivity. This decline was marked by substantial inter-individual variability and limited discrimination by treatment status, treatment response, or clinical factors such as diabetes, which together severely constrain clinical utility. Accordingly, future research should prioritize overcoming current assay limitations by minimizing technical variability and identifying high-precision biomarkers capable of reliably informing clinical and public health decision-making.

## Data Availability

The raw data supporting the conclusions of this article will be made available by the authors, without undue reservation.
