# Supplementary material for: Longitudinal dynamics and treatment-associated determinants of interferon-gamma responses in tuberculosis: a 10-year retrospective QuantiFERON-TB Gold study
Source: Front Cell Infect Microbiol. 2026 Aug 5;16:1823104. doi: 10.3389/fcimb.2026.1823104 (PMC13485823; doi:10.3389/fcimb.2026.1823104)
Supplement: Supplementary file 1 [file DataSheet1.docx]

Supplementary Material

# 1 Supplementary Figures

**Supplementary Figure 1.** Temporal trends of ∆IFN-γ levels between active and inactive tuberculosis subgroups: scatter plot and moving average curves (window size = 40)

**Supplementary Figure 2.** Discriminatory capacity of ΔIFN-γ levels between untreated and treated active tuberculosis subgroups by receiver operating characteristic (ROC) curve analysis.

**Supplementary Figure 3.** Discriminatory capacity of ΔIFN-γ levels between therapy-converted and treatment-refractory subgroups by receiver operating characteristic (ROC) curve analysis.

**Supplementary Figure 4. Distribution and group comparisons of annual IFN-γ slope**. (A) Density distribution of annual IFN-γ slope with mean and median indicated. (B) Boxplots of annual IFN-γ slope across different follow-up time intervals. (C) Comparison of annual IFN-γ slope between untreated and treated active TB patients. (D) Comparison of annual IFN-γ slope between therapy-converted healed and treatment-refractory subgroups.

**Supplementary Figure 5.** Temporal trends of ∆ IFN-γ levels between diabetic and non-diabetic subgroups: scatter plots and moving average curves (window size = 40)

**Supplementary Figure 6.** Quantitative analysis of IFN-γ levels in non-diabetic and diabetic groups. (A) Paired comparison of quantitative IFN-γ levels by Wilcoxon signed-rank test. (B) Comparison of ΔIFN-γ levels between non-diabetic and diabetic groups.

# 2 Supplementary Tables

**Supplementary Table 1.** Linear mixed-effects model results by different treatment definitions

| **Variable** | **Model 1: Treatment impact (n=353)** | **Model 2: Treatment response (n=213)** |
| --- | --- | --- |
|  | **Coefficient (95% CI) / p-value** | **Coefficient (95% CI) / p-value** |
| **Treatment × Time interaction** | **−0.889 (−1.727, −0.051) / 0.038** | **−0.641 (−1.551, 0.269) / 0.167** |
| Treatment | 0.572 (−0.340, 1.484) / 0.219 | 0.219 (−0.900, 1.337) / 0.701 |
| Visit (Post- vs pre-visit) | −0.408 (−1.131, 0.315) / 0.268 | −1.361 (−1.943, −0.779) / <0.001 |
| Sex | 0.817 (0.040, 1.593) / 0.039 | 0.934 (−0.105, 1.974) / 0.078 |
| Age | −0.045 (−0.065, −0.025) / <0.001 | −0.055 (−0.081, −0.030) / <0.001 |
| Extrapulmonary TB | −0.896 (−1.648, −0.145) / 0.019 | −0.737 (−1.729, 0.255) / 0.145 |
| Diabetes | 0.304 (−0.705, 1.313) / 0.555 | 0.525 (−0.863, 1.912) / 0.459 |
| Interval days | −0.0003 (−0.001, 0.0003) / 0.283 | −0.001 (−0.002, 0.000) / 0.138 |
| **Random effects** |  |  |
| Between-patient variance | 7.896 | 8.420 |
| Residual variance | 6.120 | 5.468 |
| ICC | 0.563 | 0.606 |
